# Lifestyle factors and reproductive health: taking control of your fertility

**DOI:** 10.1186/1477-7827-11-66

**Published:** 2013-07-16

**Authors:** Rakesh Sharma, Kelly R Biedenharn, Jennifer M Fedor, Ashok Agarwal

**Affiliations:** 1 Center for Reproductive Medicine, Cleveland Clinic, 9500 Euclid Avenue, Cleveland, OH, USA

## Abstract

Approximately 10 to 15% of couples are impacted by infertility. Recently, the pivotal role that lifestyle factors play in the development of infertility has generated a considerable amount of interest. Lifestyle factors are the modifiable habits and ways of life that can greatly influence overall health and well-being, including fertility. Many lifestyle factors such as the age at which to start a family, nutrition, weight, exercise, psychological stress, environmental and occupational exposures, and others can have substantial effects on fertility; lifestyle factors such as cigarette smoking, illicit drug use, and alcohol and caffeine consumption can negatively influence fertility while others such as preventative care may be beneficial. The present literature review encompasses multiple lifestyle factors and places infertility in context for the couple by focusing on both males and females; it aims to identify the roles that lifestyle factors play in determining reproductive status. The growing interest and amount of research in this field have made it evident that lifestyle factors have a significant impact on fertility.

## Background

It has been estimated that 7.4% of women and their husbands in the United States are infertile [1] and that the number of infertile people in the world may be as high as 15%, particularly in industrialized nations [2]. Decreasing the number of people affected by infertility has become a top priority for many health organizations, including Healthy People 2020 [3]. Lifestyle factors can be modified to enhance overall well-being and they are ultimately under one’s own control. They play a key role in determining reproductive health and can positively or negatively influence fertility.

The goal of this review is to demonstrate the potential effects of multiple lifestyles on reproductive health for both men and women. The review focuses primarily on modifiable lifestyles including the age when starting a family, nutrition, weight management, exercise, psychological stress, cigarette smoking, recreational drugs use, medications, alcohol use, caffeine consumption, environmental and occupation exposure, preventative care, clothing choices, hot water, and lubricants. While many aspects of life are not modifiable, lifestyles may be changed.

### The reproductive timeline

The age of a man or woman is a factor among others that can affect fertility. Due to pursuit of education and other factors, many couples are choosing to delay child-bearing. Fertility peaks and then decreases over time in both men and women, thus the reproductive timeline may be one aspect to consider when determining the ideal time to start a family. As men age, testosterone levels begin to decrease and hypogonadism results. However, if testosterone is used to treat hypogonadism, it can suppress spermatogenesis [4]. Semen parameters also begin a steady decline as early as age 35 [5]; semen volume and motility both decrease and morphology may become increasingly abnormal [4,6]. After the age of 40, men can have significantly more DNA damage in their sperm, as well as decline in both motility (40%) and viability (below 50%) (n = 504, p < 0.001) [7]. There may also be an increase in time to pregnancy with an increase in male age [8]. Hassan and Killick reported that when men were over the age of 45, their partner’s relative risk of an increase in time to pregnancy over one year increased to 4.6, and over two years increased to 12.5 (n = 1832, CI = 24.5-38.1) [9]. The authors also noted that the older population tended to consume more alcohol, have intercourse less often, had longer contraceptive usage, and smoked less cigarettes which could have been confounding factors. Another study found that there are also exponentially fewer infants born to fathers ≥35 to 39 years of age and older compared to younger age groups even when controlling for female age (n = 122,061) [10].

The reproductive timeline for women is complex. A woman is born with all the oocytes she will ever have, and only 400–500 are actually ovulated [6]. As the number of oocytes decline, a woman’s menstrual cycle shortens, infertility increases, and menstrual irregularity begins 6–7 years before menopause. Increasing age increases a woman’s time to pregnancy. When under the age of 30, a woman’s chances of conceiving may be as high 71%; when over 36, it may only be 41% [8]. The chances of becoming pregnant and being able to maintain a pregnancy are also affected. Matorras et al. reported that in a population of women, the number of infants born begins to exponentially decrease after the age bracket of 35–39 (n = 89,287) [10]. The odds of becoming pregnant and maintaining a pregnancy are believed to be connected to numerous factors, including euploidy. Euploidy has been found to be inversely correlated with female age (P < .01; n = 544) [11]. Another study reported that the rate of aneuploidy for women over 35 was 45.7% versus 34.8% for women under 35 (n = 352, p = .018) [12]. In comparison, Munné et al. reported the rates of euploidy decrease 50% for women under 35, decrease 40% for women between the ages of 35 and 40, and decrease 33.3% for women over 40 [13]. In addition, chromosomal abnormalities and aneuploidy may increase the risk of spontaneous abortion and implantation loss with increasing age [4,6]. Overall, women’s fertility is significantly lower in the 30s and 40s [6].

### Impacts of diet and exercise

#### Nutrition

Eating a healthy and varied diet may be a key part of maintaining good overall health. However, there are certain vitamins and food groups that could have a greater impact on reproductive health than others.

Aspects of a male’s diet may have an impact on his fertility. Consuming a diet rich in carbohydrates, fiber, folate, and lycopene [14] as well as consuming fruit (OR 2.3) and vegetables (OR 1.9) [15] correlates with improved semen quality. Consuming lower amounts of both proteins and fats were more beneficial for fertility [14]. Another potential benefit could be antioxidants, which play a pivotal role in the body by scavenging reactive oxygen species (ROS). Reactive oxygen species or ROS are a collection of free radicals and non-radical derivatives of oxygen such as superoxide anion (O_2_^•^ -), hydrogen peroxide (H_2_O_2_), hydroxyl radical (OH^•^). This category also includes free radicals derived from nitrogen called reactive nitrogen species such as: nitric oxide (NO^•^), nitric dioxide (NO_2_^•^), peroxynitrite (ONOO^-^). Collectively they are termed as reactive oxygen species. These are by-products of cellular respiration that are necessary for certain cellular activity, including sperm capacitation; however, an overabundance of ROS may compromise sperm function, including sperm motility, altering DNA and decreasing membrane integrity [16]. Antioxidants are molecules such as albumin, ceruloplasmin, and ferritin; and an array of small molecules, including ascorbic acid, α-tocopherol, β-carotene, reduced glutathione, uric acid, and bilirubin or enzymes superoxide dismutase, catalase, and glutathione peroxidase. Antioxidants help remove the excess ROS in the seminal ejaculate and assist in the conversion of ROS to compounds that are less detrimental to cells. If there is more ROS than the local antioxidants can remove, it results in oxidative stress. Oxidative stress can result in sperm protein, lipid and DNA damage and sperm dysfunction [16]. However, there have been some disputes when it comes to research outcomes. Mendiola et al. demonstrated that vitamin C, but neither vitamin E nor selenium, had significant effects on semen quality (n = 61, p < 0.05) [14]. A high amount of antioxidants has been demonstrated to increase semen quality, compared to low or moderate amounts [17]. Another study reported that vitamin E and selenium decreased levels of malondialdehyde (MDA), a marker for damage done by reactive oxygen species, more so than did vitamin B [18]. Suleimen reported that Vitamin E decreased MDA levels, increased spermatozoa motility, and led to 21% couples conceiving over a 2.5 year period versus no conceptions in men who took a placebo (n = 52) [19]. An article reviewing previous studies on antioxidants concluded almost every study conducted pertaining to DNA damage and oxidative stress revealed that antioxidants caused significant improvement, particularly in asthenospermic patients [20]. A Cochrane review including 34 studies, determined that men who use oral antioxidants had a significant increase in live birth rate (OR 4.85; CI 1.92-12.24; P = 0.0008; n = 214) when compared to control [21]. Antioxidants were also associated with a significant increase in pregnancy rate when compared to control (OR 4.18; CI 2.65-6.59; P < 0.00001; n = 964) [21].

A woman’s diet may ultimately affect her fertility, particularly ovulation. Overall, replacing carbohydrates with animal protein was demonstrated to be detrimental to ovulatory fertility (OR 1.18) [22]. Adding just one serving of meat was correlated with a 32% higher chance of developing ovulatory infertility, particularly if the meat was chicken or turkey [22]. However, replacing carbohydrates with vegetable protein demonstrated a protective effect (OR 0.5) [22]. Choosing trans fats in the diet instead of monounsaturated fats has been demonstrated to drastically increase the risk of ovulatory infertility (RR 2.31) [23]. Consuming trans fats instead of carbohydrates correlated with a 73% increase in risk of ovulatory disorder (RR 1.73) [23]. The use of multivitamins and supplements also has an effect. Women who take multivitamins may be less likely to experience ovulatory infertility; women who take six or more tablets had the lowest relative risk for infertility (RR 0.59) followed by women who took three to five (RR 0.69), and two or less (RR 0.88) [24]. Chavarro et al. found that women with high “fertility diet” scores emphasized by a higher monounsaturated to trans-fat ratio, vegetable over animal protein, high-fat over low-fat dairy, a decreased glycemic load, and an increased intake of iron and multivitamins had lower rates of infertility due to ovulation disorders (p < 0.001) [25].

#### Weight

An individual’s weight is often associated with his or her eating habits and amount of activity. Body mass index (BMI) is reported as a number. If it is below 18.5 it is considered underweight, between 18.5 and 24.9 is normal, above 25 is overweight, and over 30 is considered obese [26]. Body weight can have significant effects on health, including cardiovascular disease, diabetes, and infertility [27].

#### Obesity

The obesity epidemic has recently become is a serious issue, particularly in industrialized nations. The goal set by Healthy People 2010 of reducing obesity in the United States to 15% was not met [28]. In fact, adult obesity increased to 35.7% in 2010 [29]. The rising number of obese individuals may be due in part to an energy-rich diet as well as insufficient physical exercise [30]. In addition to other potential health risks, obesity can have a significant impact on male and female fertility.

The proportion of men over 20 years of age in the U.S. that are obese has risen to 35.5% [29]. BMI may be a significant factor in fertility, as an increase in BMI in the male by as little as three units can be associated with infertility (OR 1.12) [31]. Obese men are three times more likely to exhibit a reduction in semen quality than men of a normal weight [32]. Several studies have demonstrated that an increase in BMI is correlated with a decrease in sperm concentration [33,34], and a decrease in motility [35]. Overweight men have also been found to have increased DNA damage in sperm [36,37].

A relationship also exists between obesity and erectile dysfunction (ED). Corona et al. reported that 96.5% of men with metabolic syndrome presented with ED (n = 236) [38]. ED may be the consequence of the conversion of androgens to estradiol. The enzyme aromatase is responsible for this conversion, and is found primarily in adipose tissue [39]. As the amount of adipose tissue increases, there is more aromatase available to convert androgens, and serum estradiol levels increase [36,39]. Other hormones including inhibin B and leptin, may also be affected by obesity. Inhibin B levels have been reported to decrease with increasing weight, which results in decreased Sertoli cells and sperm production [40]. Leptin is a hormone associated with numerous effects including appetite control, inflammation, and decreased insulin secretion [41]. A study conducted in mice demonstrated that leptin was nearly five times higher in obese mice than lean mice, and that the higher leptin levels corresponded to five times lower fertility in the obese mice [41]. It was also noted that there was a down regulation of the leptin receptors located on the testes, possibly indicating that leptin resistance could play a role in male infertility [41].

In 2010, 35.8% of women in the U.S. over the age of 20 were considered obese [29]. Women with a BMI over 30 have longer time to pregnancy than women who have a BMI between 20 and 25, although this trend was not significant, and the study was conducted via a questionaire (n = 2,472) [8]. In a systematic review, Boots & Stephenson reported a miscarriage rate of 10.7% in women with a normal BMI, which was significantly lower than that of 13.6% in obese women (OR: 1.31; 95% CI 1.18-1.46) [42]. Furthermore, obese women had a higher rate of recurrent, early miscarriage compared to non-obese women. There is evidence that miscarriage in obese women may not necessarily be due to the karyotype of the developing fetus. Overweight and obese women under the age of 35 were found to have lower rates of aneuploidy, suggesting that miscarriage may be due to other influences such as endometrium receptiveness [12,43]. Additionally, Bellver et al. found a negative correlation between increasing BMI and implantation (r^2^ = .03, P = .008) [44]. A decreased ongoing pregnancy rate of 38.3% per cycle was also found in women who were overweight in comparison to the 45.5% in non-overweight women (n = 2656) [44]. There is speculation that these negative outcomes may be related to follicular environment, which differs in women who are obese compared to normal weight women. Some of the differences may include an increase in follicular fluid levels of insulin, lactate, triglycerides, and C-reactive protein; there may also be decreases in SHBG [45]. The negative effects of obesity on fertility in women may be reversible. Clark et al. found that after losing an average of 10.2 kg, 90% of obese previously anovulatory women began ovulating [46].

#### Eating disorders and being underweight

Obesity is not the only way in which weight can impact fertility. Men who are underweight are also at risk of infertility. Men who are underweight tend to have lower sperm concentrations than those who are at a normal BMI [36]. As the majority of the available literature focuses on the impact of obesity, more research is needed into the effects that being underweight may have on male fertility.

For women, being underweight and having extremely low amounts of body fat are associated with ovarian dysfunction and infertility [47]. Additionally, the risk of ovulatory infertility increases in women with a BMI below 17 (RR 1.6) [48]. A meta-analysis of 78 studies, which included 1,025,794 women, found that underweight women had an increased risk of pre-term birth (RR 1.29) [49]. Eating disorders such as anorexia nervosa are also associated with extremely low BMI. The lifetime prevalence of anorexia nervosa in women is 0.9%, with the average age of onset being 19 years old [50]. Although relatively uncommon, eating disorders can negatively affect menstruation, fertility, and maternal and fetal well-being [51]. It was found that among infertile women suffering from amenorrhea or oligomenorrhea due to eating disorders, 58% had menstrual irregularities (n = 66) [51]. Freizinger et al. reported 20.7% of infertile women seeking intra uterine insemination (IUI) had been diagnosed with an eating disorder, suggesting that women with history of eating disorders may be at a higher risk for infertility [52].

#### Exercise

A healthy amount of exercise in men can be beneficial. Physically active men who exercised at least three times a week for one hour typically scored higher in almost all sperm parameters in comparison to men who participated in more frequent and rigorous exercise (n = 45) [53]. Moderately physically active men had significantly better sperm morphology (15.2%), the only ones to be ranked above Kruger’s strict criteria in comparison to the men who played in a competitive sport (9.7%) or were elite athletes (4.7%) (P < .001). Other parameters including total sperm number, concentration, and velocity also showed a similar trend but were not nearly as marked [53]. Bicycling more than five hours per week has been demonstrated to have a negative correlation with both total motile sperm counts (OR 2.05) and sperm concentration (OR 1.92) [54]. Diet combined with exercise in obese male rats has been shown to increase both sperm motility (1.2 times) and sperm morphology (1.1 times), and to decrease both sperm DNA damage (1.5 times) and reactive oxygen species (1.1 times) (n = 40; P < .05) [55].

Physical activity has been shown to confer a protective effect on fertility when coupled with weight loss in obese women [46]. However, excessive exercise can negatively alter energy balance in the body and affect the reproductive system [56]. When energy demand exceeds dietary energy intake, a negative energy balance may occur and may result in hypothalamic dysfunction and alterations in gonadotropin-releasing hormone (GnRH) pulsality, leading to menstrual abnormalities, particularly among female athletes [57]. Increased frequency, intensity, and duration of exercise were found to be significantly correlated with decreased fertility in women, including an OR of 3.5 for infertility in women who exercised every day (n = 24,837) [58]. A study examining 2,232 women undergoing in vitro fertilization (IVF) found that women who engaged in cardiovascular exercise for 4 hours or more per week for as little as one year prior to the treatment had a 40% decrease in live birth rate (OR .6; 95% CI .4-.8), as well as higher risks of cycle cancellation (OR 2.8; 95% CI 1.5-5.3) and implantation failure (OR 2.0; 95% CI 1.4-3.1) [59]. Wise et al. also found a significant positive dose–response relationship between vigorous activity and time to pregnancy [60]. However, moderate physical activity was determined to be weakly correlated with increases in fecundity, independent of BMI.

### Psychological effects

Stress is a prominent part of any society, whether it is physical, social, or psychological. Infertility itself is stressful, due to the societal pressures, testing, diagnosis, treatments, failures, unfulfilled desires, and even fiscal costs with which it is associated [61].

Males who experienced more than two stressful life events before undergoing infertility treatment were more likely to be classified below WHO standards for sperm concentration, motility, and morphology [62]. In a study including 950 men conducted by Gollenberg et al., stress such as a job, life events, and even social strain were seen to have a significant impact on sperm density (log scale, β = −0.25; CI −0.38 to −0.11), total sperm counts (log scale, β = −0.30; CI −0.45 to −0.15), forward motility (OR 1.54; 95% CI 1.04-2.29), and morphology (OR 1.93; 95% CI 1.02-3.66) [63]. Semen parameters may potentially be linked to stress. Stress and depression are thought to reduce testosterone and luteinizing hormone (LH) pulsing [62,64], disrupt gonadal function [64], and ultimately reduce spermatogenesis and sperm parameters. It has yet to be determined if depression causes low testosterone, or if low testosterone can cause depression [65]. Although there appears to be a relationship between stress and infertility, it is uncertain which is the cause and which is the effect. The perceived stress of providing a semen sample was reported to be negatively linked to overall semen quality with a 39% decrease in sperm concentration, 48% decrease in motility, and worse overall semen parameters on the day of oocyte retrieval, although there was no change in either volume or morphology [66,67].

Stress can increase after diagnosis of infertility, follow-up appointments, and failed IVF treatments [65]. When men present to fertility clinics, 10% met the criteria for having an anxiety disorder or depression, the latter being more common [66]. Coping with various life styles also affect fertility. It was reported that actively coping with stress, such as being assertive or confrontational, may negatively impact fertility [69,70], by increasing adrenergic activation, leading to more vasoconstriction in the testes. This vasoconstriction results in lower testosterone levels and decreased spermatogenesis. While men are not often thought to report their anxiety or sexual stress, the link between anxiety and sexual stress was surprisingly strong [70]. Decreased stress levels have been associated with improvements in fertility. In one study, higher ranks in the WHO (five) Well-Being Index correlated with higher sperm concentrations [71] for each successive gain in rank, an increase in concentration of 7.3% was observed.

Physical stress has been implicated in influencing female fertility. Women who had a job and worked more than 32 hours a week experienced a longer time to conception compared to women who worked 16 to 32 hours a week [8]. Psychological stress, such as anxiety disorder or depression, affects 30% of women who attend infertility clinics, possibly due in part to infertility diagnosis and treatments [68,72]. However, this rate is not any higher than women who attend a gynecologist, but it is significantly higher than women in their second trimester of pregnancy. Only one fifth of women participating in this study were actively seeking counseling.

Receiving instruction on how to deal effectively or merely receiving support made a significant difference for women undergoing fertility treatment. There was a higher conception rate for women who were part of a cognitive behavioral intervention group (55%) or a support group (54%) than for those women who were not receiving any intervention (20%) [73]. Women who receive support and counseling may reduce their anxiety and depression levels, and increase their chances of becoming pregnant [74]. Positive moods correlated with increased chances of delivering a live baby while higher levels of anxiety increased chances of stillbirth [75]. Fertilization of oocytes also decreased when stress increased. A possible explanation for these associations may lie in stress hormone levels. One study reported that alpha amylase, but neither cortisol nor adrenalin, negatively correlated with fertility, and that the chances of conceiving in the short time period surrounding ovulation decreased [76]. Althoughthe mechanisms by which alpha amylase may decrease fertility are unknown, it is hypothesized that catecholamine receptors could alter the blood flow in the fallopian tubes [76].

### Recreational and prescription substances

#### Cigarette smoking

While it is well documented that cigarette smoke contains over 4,000 chemicals [77] and is associated with a number of potential health complications such as cardiovascular disease, more research is needed to establish a link to infertility. It is estimated that 35% of reproductive-aged males smoke [78]. Men who smoke before or during attempts to conceive risk decreasing their fertility (OR 1.6) in comparison to non-smokers [79]. Men who smoke tend to have a decrease in total sperm count, density [63], motility [80,81], normal morphology [63,81], semen volume [63], and fertilizing capacity [82]. One study, using a procedure involving hyaluronan (HA)-coated slides, found that sperm that were of a normal motility and morphology were positively correlated with high HA binding; the study determined that men who smoked had decreased HA binding, indicating that the sperm characteristics were below normal [83]. Calogero et al. concluded from their study that smoking could reduce the mitochondrial activity in spermatozoa, and lead to a decreased fertilization capacity [80]. Guar et al. reported that only 6% of 100 smokers participating in their study were classified as normozoospermic while 39% of light smokers, 19.2% of moderate smokers, and no heavy smokers experienced isolated asthenozoospermia [84]. Both moderate and heavy smokers in this study experienced astheno-, oligo-, and teratozoospermia simultaneously. Smoking also can impact DNA integrity of the sperm, with several studies noting an increase in DNA damage [80,85-88]. Saleh et al. attributed the increase in DNA damage to increased amounts of seminal leukocytes, which may have increased ROS generation to 107% [87,89]. The exact mechanism by which leukocytes and ROS affect fertility remains uncler, though it is hypothesized to be linked with the inflammatory response induced by the metabolites of cigarette smoking [87]. In addition, total antioxidant capacity (TAC) was not reduced in smokers in this study [87], contrary to other reports [90,91]. Endocrine function may also be affected by smoking, as increases in serum levels of both FSH and LH and decreases in testosterone have been reported [74].

Among women who are of reproductive age, 30% are smokers [78]. Augood et al. determined that women who smoked had a significantly higher odds ratio of infertility (OR 1.60; 95% CI 1.34-1.91), in comparison to non-smokers [79]. The reductions in fertility among female smokers may be due to decreases in ovarian function and a reduced ovarian reserve. Sharara et al. found that the incidence of reduced ovarian reserve was significantly higher in women who smoked than in age-matched non-smokers (12.31% and 4.83%, respectively), and that these women had similar fertilization and pregnancy rates [92]. This suggests that ovarian reserve may be the primary mechanism by which smoking affects fertility in women [92]. Disruption of hormone levels may also be a possible mechanism. Women who smoked 10 or more cigarettes per day were found to have a 30-35% increase in urinary FSH levels at the time of cycle transition; and women who smoked 20 or more cigarettes per day had lower luteal-phase levels of progesterone [93]. These disruptions in endocrine function could contribute to the menstrual dysfunction and infertility observed in female smokers. The uterine tube and uterus may also be targets of cigarette smoke. Chemicals in cigarette smoke may impair oocyte pick-up and the transport of fertilized embryos within the oviduct, leading to an increased incidence of ectopic pregnancies, longer times to conception, and infertility among women who smoke [94]. While using donor oocytes, Soares et al. found that women who smoked 0–10 cigarettes per day had a significantly higher pregnancy rate (52.2%) than women who smoked 10 or more cigarettes each day (34.1%), suggesting that a compromised uterine environment due to cigarette smoke was responsible for the lower pregnancy rate observed in smokers [95]. Alterations in ovarian, uterine tube, and uterine functioning, as well as disruptions in hormone levels likely contribute to the infertility observed in women who smoke.

### Drugs

#### Illicit drugs

Studies of the effects of illegal drugs on human fertility have been scarce due to ethical considerations, as well as subject to under-reporting and bias due to the characteristics of the population being studied, such as low socioeconomic status or improper prenatal care [61]. Use of illicit drugs appear to have a negative impact on fertility, though more in-depth research in this area is required to make a clear link.

Marijuana is one of the most commonly used drugs around the world [96], and it acts both centrally and peripherally to cause abnormal reproductive function. Marijuana contains cannabinoids which bind to receptors located on reproductive structures such as the uterus or the ductus deferens. In males, cannabinoids have been reported to reduce testosterone released from Leydig cells, modulate apoptosis of Sertoli cells, decrease spermatogenesis, decrease sperm motility, decrease sperm capacitation and decrease acrosome reaction [96]. Females who use marijuana are at an increased risk of primary infertility in comparison to non-users (RR 1.7; 95% CI 1.0-3.0) [97]. In women, use of marijuana can negatively impact hormonal regulation; over short periods of time, marijuana may cause a drop in the levels of luteinizing hormone, but over long periods of time, the hormone levels may remain constant due to developed tolerance [98]. Marijuana and its cannabinoids have been reported to negatively impact movement through the oviducts, placental and fetal development, and may even cause stillbirth [96-99].

Another commonly used recreational drug is cocaine, a stimulant for both peripheral and central nervous systems which causes vasoconstriction and anesthetic effects. It is thought to prevent the reuptake of neurotransmitters [100], possibly affecting behavior and mood. Long term users of cocaine claim that it can decrease sexual stimulation; men found it harder to achieve and maintain erection and to ejaculate [101]. Cocaine has been demonstrated to adversely affect spermatogenesis, which may be due to serum increases in prolactin, as well as serum decreases in total and free testosterone [102,103]. Peugh and Belenko suggest that the effects of cocaine in men depend on dosage, duration of usage, and interactions with other drugs [104]. While less is known about cocaine’s effects on females, impaired ovarian responsiveness to gonadotropins and placental abruption have both been reported [105-107].

Opiates comprise another large group of illicit drugs. Opiates, such as methadone and heroin, are depressants that cause both sedation and decreased pain perception by influencing neurotransmitters [104]. In men taking heroin, sexual function became abnormal and remained so even after cessation [108]. Sperm parameters, most noticeably motility, also decrease with the use of heroin and methadone [103,109]. In women, placental abruption with the use of heroin may also be a cause of infertility [61].

#### Prescription drugs

In general, there are more studies reviewing the effects of medication on male than female fertility. It is necessary to first determine which medications cause fertility issues, and to then determine if these effects are permanent. A study headed by Hayashi, Miyata, and Yamada investigated the effects of antibiotics, antidepressants, antiepileptics, β stimulators, H1 and H2 receptor antagonists, mast cell blockers, and sulfonylurea compounds (n = 201) [110]. Male participants were divided so one group had medication switched or stopped and the other served as the control. The intervention group improved 93% in semen quality and 85% of the group conceived in 12.5 months ± .64 months; and the control group improved 12% in semen quality and only 10% conceived. The authors suggested that this study may link certain tested medications with impaired semen qualities [110]. Additional medications and their effects on both males and females are represented in Table 1[61].

**Table 1:** Medications and their respective effects on both male and female reproductive function

**Medication**	**Effect on reproductive function**
**Anabolic Steroids**	Impairment of spermatogenesis (up to one year recovery); may cause hypogonadism through pituitary–gonadal axis
	Reversible
**Antiandrogens:**	Impairment of spermatogenesis; erectile dysfunction
Cyproterone acetate, danazol, finasteride, ketoconazole, spironolactone	Reversible
**Antibiotics:**	Impairment of spermatogenesis
Ampicillin, cephalotin, cotrimoxazole, gentamycin, neomycin, nitrofurantoin, Penicillin G, spiramycin	Reversible
**Antibiotics:**	Impairment of sperm motility
Cotrimoxazole, dicloxacillin, erythromycin, lincomycin, neomycin, nitrofurantoin, quinolones, tetracycline, tylosin	
	Reversible
**Antiepiletics:**	Impairment of sperm motility
Phenytoin	Reversible
**Antihypertensives:**	Fertilization failure
Calcium channel blockers (nifedipine)	
**Antihypertensives:**	Erectile dysfunction
Alpha agonists (clonidine), alpha blockers (prazocin), beta blockers, hydralazine, methyldopa, thiazide diuretics	
**Anti-inflammatory 5-ASA and derivatives:**	Impairment of spermatogenesis and sperm motility
Mesalazine, sulfasalazine	Reversible
**Antimalarials:**	Impairment of sperm motility
Quinine and its derivatives	Reversible
**Antimetabolites ⁄ Antimitotics:**	Arrest of spermatogenesis; azoospermia
	Irreversible
Colchicines, cyclophosphamide	
**Anti-oestrogens**	Impairment of endometrial development
Clomiphene citrate	reversible
**Anti-progestins:**	Impairment of both implantation and tubal function
Emergency contraceptive pills, progesterone-only pills	
**Antipsychotics:**	Increase prolactin concentrations that can lead to sexual dysfunction
Alpha blockers, phenothiazine, antidepressants (particularly SSRIs)	
**Antipsychotics:**	Impairment of spermatogenesis and sperm motility
Butyrophenones	Reversible
**Antischistozomal:**	Impairment of spermatogenesis and sperm motility
Niridazole	Reversible
**Corticosteroids**	Impairment of sperm concentration and motility
	Reversible
**Exogenous testosterone, GnRH analogues**	Impairment of spermatogenesis
	Reversible
**H2 blockers:**	Increase prolactin concentrations that can lead to impairment of luteal function, loss of libido, and erectile dysfunction
Cimetidine, ranitidine	
**Local anaesthetics, halothane**	Impair sperm motility
**Metoclopramide**	Erectile dysfunction
**Methadone**	Suppress spermatogenesis and sperm motility
**Non-steroidal anti-inflammatory drugs, Cox-2 inhibitors**	Impairment of follicle rupture, ovulation, and tubal function
	Reversible

### Alcohol

Many studies have been conducted on the effects of alcohol and aspects of health, including fertility. While there are studies that demonstrate the link between alcohol and infertility, it is not entirely clear what amount relates to an increased risk.

In men, alcohol consumption has been linked with many negative side effects such as testicular atrophy, decreased libido, and decreased sperm count [111-113]. One meta-analysis including 57 studies and 29,914 subjects found a significant association between alcohol and semen volume (P = .0007; I squared statistics (I^2^) n = 35) [63]. A link between alcohol and sperm morphology has also been found. Very few men who are classified as alcoholics were normozoospermic with only 12% of men in one study being designated as such; most alcoholics were found to be teratozoospermic, with 73% of heavy drinkers and 63% moderate drinkers falling in this category (n = 100; P = .0009) [84]. In addition, oligozoospermia was another common classification for heavy drinkers (64%) in this study (P = 0.0312). Alcohol seems to have a large impact on both sperm morphology and sperm motility [84]. While alcohol may have effects on sperm morphology, there is little conclusive evidence linking alcohol with oxidative stress, and infertility. Oxidative stress has been found to systemically increase with alcohol consumption [114,115], but there is not yet a clear link between sperm oxidative stress and alcohol [91].

Women who drink large amounts of alcohol have a higher chance of experiencing an infertility examination than moderate drinkers (RR = 1.59, CI 1.09 –2.31) in comparison to those who consumed low amounts, who had a decreased chance of experiencing an infertility examination (RR 0.64; CI 0.46-0.90) (n = 7,393) [116]. A common result of drinking is a hangover. Women who experienced hangovers were more likely to be infertile than women who did not experience hangovers [117], suggesting that the amount of alcohol consumed does matter. While it is clear alcohol can have an impact, the amount it takes to negatively influence reproductive function is not clear as there is no standard “drink”. Amounts of alcohol ranging from one drink a week to 5 units a day can have various effects including increasing the time to pregnancy (P = .04; 95% CI .85-1.10) [8], decreasing probability of conception rate by over 50% [118] and decreasing implantation rate, increasing both the risk of spontaneous abortion (OR 4.84) [119] and of fetal death [120], and causing anovulation, luteal phase dysfunction, and abnormal blastocyst development [121]. Researchers believe that these effects may be due to hormonal fluctuations including increases in estrogen levels, which reduce FSH and suppress both folliculogenesis and ovulation [116,121], but many mechanisms are still unknown.

### Caffeine

Caffeine has become an integral part of society with consumption varying from 50 mg in a 16 oz. bottle of Pepsi to 330 mg in a 16 oz. cup of Pikes Place Roast from Starbucks [122,123]. However, caffeine has been reported to have negative effects on female fertility. Caffeine has been associated with an increase in the time to pregnancy of over 9.5 months, particularly if the amount is over 500 mg per day (OR 1.45; 95% CI1.03-2.04) [124]. The negative effects that are emphasized in recent research are miscarriage, spontaneous abortion, fetal death and still birth. Women who consumed more than 100 mg of caffeine a day were more likely to experience a miscarriage (151 mg-300 mg: OR 3.045; 95% CI: 1.237–7.287, p = 0.012; over 300 mg; OR 16.106; 95% CI 6.547–39.619, p < 0.00; n = 312) [125] or spontaneous abortion [126,127]. The karyotypes of those spontaneously aborted fetuses in women who consumed more than 500 mg of caffeine a day were also more likely to be normal (n = 1,515; OR 1.4; 95% CI .5-3.7) [126], indicating that spontaneous abortions may not be due to genetic defects, but perhaps an unknown mechanism triggered by caffeine. Greenwood et al. demonstrated that caffeine consumption during the first trimester is related to both miscarriage and still birth (n = 2,643) [128]. The women who miscarried or had a still birth in their study had an average of 145 mg of caffeine per day (95% CI 85–249); and women who had live births consumed an average of 103 mg per day (95% CI 98–108), indicating that there may be a narrow window for caffeine to impact fertility. Women who consumed more than 375 mg of caffeine a day had an odds ratio for spontaneous abortion higher than women who had fewer than 200 mg a day (330 subjects, 1168 controls; OR 2.21; CI 2.53-3.18) [119]. In 2003, Wisborg et al. found that after adjusting for smoking and drinking, women who drank four to seven cups of coffee had nearly an 80% increase in chance of still birth, and those who consumed more than 8 cups of coffee a day had nearly a 300% increase (OR 3.0; 95% CI 1.5-5.9; n = 18.478) [129]. Another study including over 88,000 women demonstrated that if over 8 cups of coffee were consumed, the risk for fetal death increased [130].

### Environmental and occupational exposures

Many potential threats to reproductive health are encountered in every-day life through biological (viruses), physical (radiation), and toxic (chemicals) sources [131]. While the human body has defenses to protect itself, these threats can still influence one’s health through inhalation, ocular and dermal contact, ingestion, and vertical and horizontal transfer [132]. These hazards may also have negative ramifications for fertility.

#### Air Pollution

Air pollution is the release of pollutants such as sulfur dioxides, carbon monoxide, nitrogen dioxide, particulate matter, and ozone into the atmosphere from motor vehicle exhaust, industrial emissions, the burning of coal and wood, and other sources [132,133]. While air pollution has received a tremendous amount of attention in the past few decades for many health reasons, its effects on fertility are less well-known.

There have been reports of air pollution and its impacts on male fertility. Several studies have been conducted in the Czech Republic regarding men living in two different locations, one more polluted than the other [134,135]. Men who are exposed to higher levels of air pollution were more likely to experience abnormal sperm morphology, decreased motility, and an increased chance of DNA fragmentation (n = 48 or 408 respectively). There was also a significant negative correlation found between sperm concentration and the amount of ozone to which a man was exposed (n = 5134) [136].

Negative reproductive side effects of air pollution on women can include preterm delivery [132,137], miscarriage, stillbirth, spontaneous abortion, and fetal loss [132]. Many times when fetal loss occurred, there were malformations within the fetal reproductive tract.

#### Heavy metals

Heavy metals include metals such as lead, mercury, boron, aluminum, cadmium, arsenic, antimony, cobalt, and lithium. Only a few such heavy metals have been researched in connection to reproductive function. Lead, which is commonly found in batteries, metal products, paints, ceramics, and pipes, is one of the most prominent heavy metals. Lead interrupts the hypothalamic-pituitary axis and has been reported to alter hormone levels [132,138], alter the onset of puberty, and decrease overall fertility [132]. Lead may alter sperm quality in men, and cause irregular menstruation, induce preterm delivery, and cause miscarriage, stillbirth, and spontaneous abortion in women [132]. Mercury is commonly found in thermometers, batteries, and industrial emissions. Mercury concentrations increase in the food chain, resulting in bioaccumulation that can negatively impact reproduction in humans who consume food, usually tainted seafood [132]. Ultimately, mercury can disrupt spermatogenesis and disrupt fetal development [138]. Boron is another heavy metal that is used in the manufacturing of glass, cement, soap, carpet, and leather; its effects on the hypothalamic-pituitary axis are comparable to lead [138]. While there is not much research on cadmium, it has been shown experimentally to cause testicular necrosis in mice, as well as marked changes in libido and infertility [139].

#### Pesticides, endocrine disruptors, and other chemicals

Many of the chemicals used world-wide in today’s society, including pesticides and endocrine disruptors, among others, may have various damaging effects on the reproductive health of both men and women. Mimicking natural hormones, impeding normal hormone activity, and varying regulation and function of the endocrine system are a few of the many ways that endocrine disruptors influence one’s body [138]. Numerous studies have reported negative effects of a variety of chemicals on reproductive health [132,138,140-144] (Table 2).

**Table 2:** Chemicals and their respective effects on both male and female reproductive function

**Chemical**	**Possible reproductive effects**	**Sources included**
**BPA**	Inhibits binding to androgen receptor, decreased semen quality, erectile dysfunction, chromosomal abnormalities in oocyte, recurrent miscarriage,	[132,140,144]
**Disinfection by-products**		
**Organochemicals and Pesticides** e.g. DDT, DDE, Methoxyclor	Change in hormone levels, irregular menstruation, decreased fertility, decreased semen quality, chromosomal abnormalities in sperm, altered histology of testes, decreased libido, fetal loss, miscarriage	[132,138,142-144]
**Dioxins**	Changes in hormone levels, altered puberty, altered start of menarche, endometriosis, decreased fertility, fetal loss	[132,143]
**Phthalates**	Decreased semen quality, oligozoospermia, earlier menarche, altered menstrual cycle, infertility	[132,144]
**Solvents**	Change in hormone levels, decreased semen quality, irregular menstruation, decreased fertility, miscarriage, fetal loss	[132]

#### Occupation and hobbies

Both men and women can be exposed to chemicals and other materials that may be detrimental to their reproductive health while on the job. Heavy metals and pesticides, as outlined in Table 2, have many negative side effects, particularly for those who work around them. Men working in agricultural regions and greenhouses which use pesticides have higher concentrations of common pesticides in their urine [145], overall reduced semen parameters [146], oligozoospermia [15], lower sperm counts [147], and sperm concentrations decreased by as much as 60% [148]. Organic solvents may also prove detrimental. Men who work with these substances often experience indirect consequences with their female partner having decreased implantation rates (n = 726) [149]. Welding is another possible source of occupational exposure, and plays a role in reduced reproductive health [15,150]. There are also consequences for working in factories that manufacture chemicals and heavy metals. Factories that produce batteries where workers are exposed to lead may have negative impacts on reproductive capabilities, including asthenospermia and teratospermia (n = 150) [151]. Hobbies, while not often associated with excessive amounts of exposure, may be just as damaging as manufacturing. Gardeners may be in contact with pesticides [150]; crafters making jewelry, ceramics, and even stained glass may come in contact with lead [132]; painters may also come in contact with lead-based paints [150]. Whether it is manufacture or hobby, using any kind of heavy metal or pesticide likely will result in some exposure, and possibly reduce fertility.

#### Radiation

Exposure to various kinds and amounts of radiation can have lasting effects in humans. Radiation that is in the form of x-rays and gamma rays can be devastating to the sensitive cells of the human body, including germ and Leydig cells. The damage done depends on the age of the patient and dose, and ultimately can result in permanent sterility [2,152].

The incredible convenience of the cell phone has dramatically increased its usage in the last decade. However, it does not come without negative effects. There have been an increasing number of studies demonstrating negative effects of the radiofrequency electromagnetic waves (RFEMW) utilized by cell phones on fertility. Cell phone usage has been linked with decreases in progressive motility of sperm [153], decreases in sperm viability [153,154], increases in ROS [154], increases in abnormal sperm morphology, and decreases in sperm counts [153]. One study evaluating 52 men demonstrated that men who carried a cell phone around the belt line or hip region were more likely to have decreased sperm motility (49.3 ± 8.2%) compared to men who carried their cell phones elsewhere or who did not carry one at all (55.4 ± 7.4%; P < .0001) [155]. Link between cell phones and fertilization capacity. Falzone et al. reported that when exposed to RFEMW, sperm head area significantly decreased from 18.8 ± 1.4 μm^2^ to 9.2 ± .7 μm^2^ and acrosomal area significantly decreases from 21.5 ± 4% to 35.5 ± 11.4% (P < .05) [156]. In addition, Falzone et al. found the mean number of sperm binding to the zona was significantly less in the exposed group (22.8 and 31.8 respectively) [156]. While amount of research demonstrating negative effects of cell phone usage and fertility grows, there can be no clear conclusion as no standard for analyzing cell phone effects is available and many studies have limitations [157,158]. Another aspect to consider is the effect of text-messaging on the body, as it is becoming more prevalent in respect to making phone calls. While technology quickly advances, research lags behind [159], providing the opportunity for unforeseen damage to occur.

### Preventative care

#### Contraceptive use

While contraceptives are often associated with preventing pregnancy, several studies have demonstrated that both condom usage and oral contraceptives can preserve fertility in women [8,160]. In 2010, Revonta et al. concluded that infertile women used less oral contraception [117]; women who considered themselves infertile might be less inclined to use contraceptives [8]. Contraceptives are believed to reduce the chances of contracting a sexually transmitted infection, thus reducing infertility. Contraceptives also may decrease time to conception. In one study, condom users had shorter time to conception compared to oral contraceptive users; oral contraceptive users in turn had shorter time to conception than those women not using any contraceptives [117]. In addition, oral contraceptives were demonstrated to have positive effects on the prevention and management of endometriosis and pelvic inflammatory disease [117]. This evidence suggests that contraceptives may increase a woman’s fertility, lending to the overall fertility of the couple.

#### Doctor visits

Scheduling regular doctor appointments may be beneficial for fertility. Males tend to not seek medical treatment for sexual dysfunctions or infections. It was reported that when men experience sexual problems, only 10.5% seek help (n = 11,161) [157]. When the problems become on-going, 20.5% of men turn to health care professionals [161]. Mercer et al. concluded that the low amount of males seeking treatment is most likely due to lack of awareness of treatment and guidance [161].

For women, visiting the gynecologist to receive an annual pap smear has been associated with being protective of fertility (n = 10,847) [160]. Kelly-Weeder and Cox also concluded from their study that when a woman reports her health status as good, she is more likely to be fertile. Both pap smears and self-reported health status may be related to better screening for disease, STI detection, more available information, and overall better access to care.

### Other factors

#### Clothing

The type of clothing a man chooses to wear, may have effects on reproductive health. Many studies have been conducted hoping to find an answer to the question of what type of clothing is best for fertility. The view that elevation of scrotal temperature negatively impacts spermatogenesis and sperm parameters is universally acknowledged [162]. But the question of whether tight-fitting underwear actually has an effect on scrotal temperature and therefore semen quality has long been debated. There have been studies that have found significantly higher temperatures with tight-fitting clothing versus loose-fitting or no clothing [163,164]. Increases in scrotal temperatures could be due to an increase in temperature of about 3.5°C of the air between the clothing and the skin in comparison the ambient air [164].

One study followed 20 participants who wore tight-fitting underwear for 6 months then switched to loose-fitting underwear for 6 months [165]. Semen samples were taken every 2 weeks for the duration of the study. While half of the participants dropped out, there was a significant 50% decrease in sperm parameters in the tight-fitting versus loose-fitting underwear, demonstrating that the effects of tight-fitting underwear reversible. In another study, men who wore tight-fitting underwear and pants had a relative risk of 2.5 of having impaired semen quality [166]. They also noted that only wearing one or the other caused an insignificant decrease in semen quality. While there are studies that conclude that the type of underwear influences scrotal temperature, there are also some that did not find significant temperature differences [167,168].

#### Hot water

Literature providing evidence that wet heat is tied to infertility is scarce. Many fertility authorities rely on the data provided from research of the effects of temperature on sperm function and then apply the idea to hot baths, jacuzzis, or saunas. One study conducted by Shefi et al. actually studied the effects of wet heat on 11 male subjects who were exposed to wet heat for greater than 30 minutes every week for at least 3 months prior to any experimentation [169]. These 11 men were then told to avoid wet heat exposure for 3 months. Three different semen samples were assessed: one from the onset of the study representing the exposed, one before 3 months into the experiment, and another at 3 to 6 months. Nearly half of the participants saw an increase in semen quality. Sperm motility saw a significant 22% increase for responders, and the improvement appeared to continue longer than 3 months (P = .02). When reviewing the non-responders, Shefi et al. found that other lifestyle factors could have accounted for the lack of semen quality increase, such as tobacco use.

#### Lubricants

Many sexually active couples choose to utilize vaginal lubricants to treat vaginal dryness and pain during intercourse [170]. While attempting to conceive, nearly 75% of participating couples reported to an internet study that they used lubricants to ease the female partner’s vaginal dryness, and 26% had claimed that they almost always used a lubricant [171]. Some non-commercial products used as lubricants include olive oil, vegetable oil, and saliva, and they have been demonstrated to negatively impact sperm function. Several products available to the public have been researched for possible effects on sperm function. A study conducted by Agarwal et al. compared Replens, Astroglide, FemGlide, K-Y Jelly, and Pre ~ Seed against a control medium [170]. In relation to the control, Astroglide, FemGlide, and Replens all significantly decreased sperm motility after 30 minutes of contact with semen (P < .01). Astroglide and Replens had a greater impact on motility in comparison to FemGlide’s. They also found that FemGlide and K-Y Jelly significantly increased sperm chromatin damage in comparison to the control medium (P < .05). While Pre ~ Seed caused an increase in chromatin damage, it was not significant.

## Conclusions

Lifestyle factors, including age when starting a family, nutrition, weight management, exercise, psychological stress, cigarette smoking, recreational and prescription drugs use, alcohol and caffeine consumption, environmental and occupational exposures, preventative care, and other behaviors are modifiable and may impact fertility.

The evidence suggests that age may play a large role in determining fertility. Attempting pregnancy before the age of 30 for women and before 35 for men may provide the highest chances of success. While it is important for one partner to consider their age, it is when both partners consider their ages together that they may be able to thoroughly increase their odds of having a successful pregnancy.

Proper nutrition, weight, and exercise may impact fertility. Though no definitive link has been drawn, choosing proper nutrition, whether it be choosing supplements or food groups, before and during attempts to conceive may be vital for improving fertility for both men and women. Men and women who are underweight or overweight are also at risk for negative side effects, including changes in hormone levels that heavily influence their fertility. Recent research suggests that weight plays an important role in fertility, and controlling and maintaining an ideal weight may provide a way for couples to increase their fertility. Exercise is suggested to be beneficial, though too much may be detrimental. Lean and underweight men or women who exercise vigorously may put themselves at risk for a decrease in fertility, thus finding a balance may provide the best chances of achieving a pregnancy.

While there are associations between psychological effects and infertility, it is hard to establish a cause-effect relationship. Tests are subjective, and there is no general consensus on how to measure psychological stress [2]. It is also worth noting that it is difficult to isolate psychological effects because subjects who are more depressed and anxious are also more inclined to participate in lifestyles that may negatively influence fertility, such as consuming alcohol [65]. Couples attempting to conceive may try relaxing and reducing exposure to stressors in an effort to increase fertility [76].

Recreational and prescription substances also appear to have significant impact on fertility. Though clear links are yet to be determined, there are negative trends, including decreased semen parameters or decreased ovarian reserve, associated with smoking and fertility. If couples are attempting to achieve a pregnancy, limiting or smoking cessation may provide more positive outcomes. It is difficult to establish a link between illegal drug use and infertility as there are many ethical issues that prevent researchers from discovering a definitive relationship. Researchers have also had issues with drawing a definitive link between infertility and prescription medication, which often have known side effects, though fertility side effects are not often a concern when prescribing. Further research on many medications and drugs is needed in order to make any recommendations. While there is evidence to support that alcohol does have an impact on fertility, it is also difficult to establish a definitive link as there is no standard “drink” or comparative way to measure alcohol consumption. Despite these drawbacks to recent research, decreasing or ceasing alcohol consumption may provide a better chance of achieving a viable pregnancy for the couple. Though caffeine appears to have a negative effect on fertility, additional research is needed to elucidate if there is a definitive relationship.

Concerning environmental exposures, assessing the exposures of each individual may be crucial to reproductive health of the couple. Eliminating every exposure is unrealistic; however, identifying, eliminating, or minimizing even one factor may have significant positive effects on fertility for both men and women.

Taking care of a current fertility problem may provide better fertility in the future. Taking preventative steps such as visiting your doctor and using contraception may help fertility. Making appointments with a doctor for both preventative measures and when problems arise may assist in increasing fertility for both men and women. In addition, using appropriate contraception may have a positive impact on a couple’s fertility. Other factors such as clothing choice, wet heat, and lubricants may also impact a couple’s fertility. Overall, while there is suggestive data, a clear negative influence of the type of underwear or clothing on semen quality has not been proven, and the overall effects on a couple’s fertility are still unknown. While there is suggestive data, there is very little actual evidence linking wet heat and fertility to suggest cessation of the use of wet heat. Research suggests that some lubricants can be beneficial for couples trying to conceive, and some lubricants may be detrimental to fertilization. Choosing an appropriate lubricant may provide the couple with improved chances of achieving a pregnancy.

The lifestyle factors discussed in the present review have the potential to impact fertility. It is important to understand the ways in which lifestyle behaviors may benefit or harm fertility in order to minimize complications and to maximize fertility outcomes. By understanding the impact of lifestyle on reproductive health, and by actively modifying lifestyle behaviors, men and women are capable of controlling their own fertility potential.

## Abbreviations

OR: Odds ratio; ROS: Reactive oxygen species; IUI: Intra uterine insemination; MDA: Malondialdehyde; RR: Relative risk; BMI: Body mass index; ED: Erectile dysfunction; SHBG: Sex Hormone-Binding Globulin; GnRH: Gonadotropin-Releasing Hormone; LH: Luteinizing Hormone; HA: Hyaluronan; TAC: Total antioxidant capacity; FSH: Follicle stimulating hormone; RFEMW: RadioFrequency ElectroMagnetic Waves; BPA: Bisphenol A; DDT: DichloroDiphenylTrichloroethane; DDE: DichloroDiphenyldichloroEthylene; 5-ASA: 5-Aminosalicylic acid.

## Competing interests

The authors declare that they have no competing interests.

## Authors’ contributions

RKS conceived the study, participated in the study design compilation of the contents and critical review of the paper. KRB and JMF were responsible for literature (Medline) search, compilation of the information, drafting and finalizing the paper. AA provided substantial contribution ranging from study idea, design, and critical review of the final paper. All authors read and approved the final manuscript.

## Authors’ information

KRB and JMF were summer interns at the Center for Reproductive Medicine, Glickman Urological & Kidney Institute, Cleveland Clinic, Cleveland, Ohio. RS is the Coordinator of the Center for Reproductive Medicine, Cleveland Clinic, Cleveland Ohio. AA is the Director of the Center for Reproductive Medicine, Cleveland Clinic, Cleveland Ohio.
